# A One-Dimensional CNN-LSTM Model for Epileptic Seizure Recognition Using EEG Signal Analysis

**DOI:** 10.3389/fnins.2020.578126

**Published:** 2020-12-10

**Authors:** Gaowei Xu, Tianhe Ren, Yu Chen, Wenliang Che

**Affiliations:** ^1^Department of Cardiology, Shanghai Tenth People’s Hospital, Tongji University School of Medicine, Shanghai, China; ^2^School of Informatics, Xiamen University, Xiamen, China; ^3^Department of Dermatology & STD, Nantong First People’s Hospital, Nantong, China

**Keywords:** convolutional neural network, electroencephalographic, epileptic seizure recognition, long short-term memory, signal analysis

## Abstract

Frequent epileptic seizures cause damage to the human brain, resulting in memory impairment, mental decline, and so on. Therefore, it is important to detect epileptic seizures and provide medical treatment in a timely manner. Currently, medical experts recognize epileptic seizure activity through the visual inspection of electroencephalographic (EEG) signal recordings of patients based on their experience, which takes much time and effort. In view of this, this paper proposes a one-dimensional convolutional neural network-long short-term memory (1D CNN-LSTM) model for automatic recognition of epileptic seizures through EEG signal analysis. Firstly, the raw EEG signal data are pre-processed and normalized. Then, a 1D convolutional neural network (CNN) is designed to effectively extract the features of the normalized EEG sequence data. In addition, the extracted features are then processed by the LSTM layers in order to further extract the temporal features. After that, the output features are fed into several fully connected layers for final epileptic seizure recognition. The performance of the proposed 1D CNN-LSTM model is verified on the public UCI epileptic seizure recognition data set. Experiments results show that the proposed method achieves high recognition accuracies of 99.39% and 82.00% on the binary and five-class epileptic seizure recognition tasks, respectively. Comparing results with traditional machine learning methods including k-nearest neighbors, support vector machines, and decision trees, other deep learning methods including standard deep neural network and CNN further verify the superiority of the proposed method.

## Introduction

Epilepsy is a neurological disorder, caused by various genetic and acquired factors, which has affected over 50 million people all over the world (Galanopoulou et al., 2012; Abraira et al., 2019; San-Segundo et al., 2019). Usually, epilepsy is caused by the abnormal activities in the brain, it leads to various symptoms, including temporary confusion, loss of consciousness or awareness, uncontrollable jerking movements and so on. Epilepsy seriously affects both the physical and mental health of patients, and in some extreme cases, it even poses a threat to patients’ life. Therefore, it is urgent and important to provide timely and effective protective measures for people with epilepsy and thus improve life quality of patients (Tsubouchi et al., 2019).

Electroencephalographic (EEG) provides a noninvasive biophysical examination method for medical experts to studying the characteristics of epilepsy, which can offer much detailed information of epilepsy patients that cannot be obtained by other physiological methods (Adeli et al., 2007; Jiang et al., 2017a,b). Traditionally, medical experts diagnose epilepsy and determine the cause of seizures through visually analyzing the EEG signal data based on their experience, which takes much time and effort. To date, there have been many attempts to automatically recognize epileptic seizure activity using advanced deep learning techniques (Adeli et al., 2007; Jiang et al., 2015; Schmidhuber, 2015; Jiang et al., 2020a,b).

In recent years, deep learning has developed tremendously and is widely used in various fields, especially in image processing and natural language processing (Acharya et al., 2013; Jiang et al., 2015; LeCun et al., 2015). Convolutional neural network (CNN), as one of the most famous deep learning models, can extract abundant features by using various filters in the convolutional layers, pooling layers, normalization layers, and fully connected layers, thereby improving the execution performance of various tasks (Radenovic et al., 2019). However, CNN cannot retain memory of previous time series patterns and thus, it is challenging for CNN to directly learn the most important and representative features from EEG biomedical signals in the form of time series. Consequently, CNN has difficulty in accurately constructing the relationship between the raw EEG signals and the epileptic seizure recognition results.

Recurrent neural network (RNN), as a specific type of neural networks, uses previous outputs as inputs and thus can remember information from the past (Choi et al., 2016; Kong et al., 2019). Recently, there have been many researches applying the RNN in the fields of natural language processing and speech recognition. Long short-term memory (LSTM) is one of RNN architectures (Hanson et al., 2016; Jiang et al., 2019) and has been widely adopted for time series processing. Its design specifically solves the gradient vanishing problem in the basic RNN and helps to learn long-term dependencies, which can acquire the temporal features of sequential data more effectively. In order to model the sequence temporally and improve the modeling capabilities of the deep neural networks, T. Sainath et al. combined the advantages of CNN and RNN to form a convolutional LSTM neural network (Sainath et al., 2015) and applied it on a variety of large vocabulary tasks, which provides a 4–6% relative improvement over an LSTM. In addition, there are also many other studies focusing on extracting temporal and spatial features by combining CNN and LSTM models, which proves the effectiveness and superiority of this solution (Jiang et al., 2019). Therefore, in this paper, in order to improve the performance of EEG signal classification, a CNN-LSTM model is proposed for features extraction of EEG signals and automatic recognition and detection of epileptic seizure (Sun et al., 2019).

The rest of this paper is organized as follows: section “Dataset Description” introduces the UCI epileptic seizure recognition data set. Section “Proposed Method” presents the proposed method, including data pre-processing, 1D CNN, LSTM structure, the 1D CNN-LSTM model, and model application. Section “Method Evaluation” presents the experimental results and provides experimental analysis. Finally, the conclusion and future works are discussed in section “Conclusion and Future Work.”

## Data Set Description

The data set used in this paper is the public UCI epileptic seizure recognition data set (Andrzejak et al., 2001). In the original data set, there are five different folders with 100 files for each folder. To be specific, each file represents a recording sample of the brain activity from one subject. In each file, there is a recording of brain activity with 4097 data points, which is sampled for 23.5 s. That is to say, there is a total of 500 subjects in this data set, each has a recording sample with 4097 data points.

The original dataset is first pre-processed by the UCI and then published online. Each sample with 4097 data points is divided into 23 data chunks and each chunk has 178 data points of 1 s. After that, the 23 data chunks are shuffled. Finally, for the 500 subjects, 11,500 time-series EEG signal data samples are obtained.

There are five health conditions in the UCI epileptic seizure recognition data set, they include one epileptic seizure condition and four normal conditions where subjects do not have epileptic seizures. The details of them are as follows:

(1) Epileptic seizure condition: the recordings of subjects who have epileptic seizures;
(2) First normal condition: the recordings of subjects who opened their eyes when they were recording the EEG signals;
(3) Second normal condition: the recordings of subjects who closed their eyes when they were recording the EEG signals;
(4) Third normal condition: the recordings of EEG signals collected from the healthy brain area of subjects;
(5) Fourth normal condition: the recordings of EEG signals collected from the tumor area in the brain of subjects.

**FIGURE 1:**
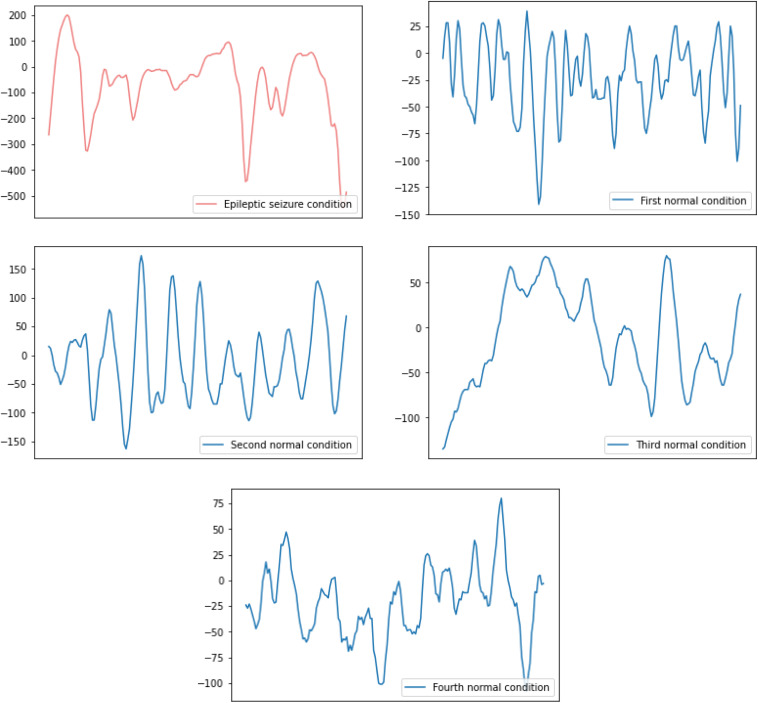
The raw EEG signal waveform of one epileptic seizure condition and four normal conditions.

The raw EEG signal data of the subjects’ five health conditions is shown in Figure 1. The difference between the raw EEG signal waveform of the epileptic seizure condition and the normal condition can be easily observed, while the difference between the raw EEG signal waveform of the different normal conditions can hardly be observed. Therefore, in this paper, binary and five-class epileptic seizure recognition tasks are both considered in order to thoroughly evaluate the performance of the proposed method.

## Proposed Method

In this section, the proposed epileptic seizure recognition method based on the 1D CNN-LSTM model is presented. Firstly, the raw EEG signal data is pre-processed; then, the 1D CNN and LSTM are introduced, respectively; finally, the 1D CNN-LSTM model is designed and applied for epileptic seizure recognition.

### Data Pre-processing

As we introduced in section “Dataset Description,” the original data set has been processed and re-structured in advance by a UCI official. Thus, in the data pre-processing process, we only normalized the EEG signal data provided by the UCI official data set before feeding it into the neural network.

### 1D CNN

**FIGURE 2:**
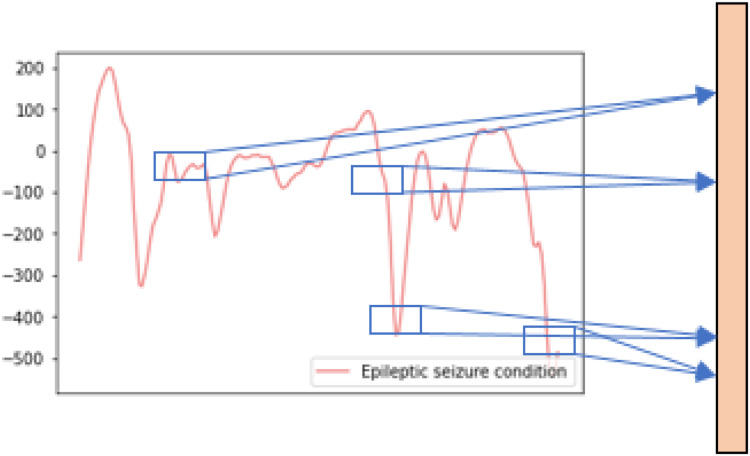
The details of the 1D convolution operation.

The 1D CNN can extract the effective and representative features of 1D time-series sequence data through performing 1D convolution operations using multiple filters. In this paper, the convolutional filters and feature maps of the 1D CNN are all one-dimensional, thus it can match the one-dimensional characteristic of raw EEG signal data, the details of the 1D convolution operation are shown in Figure 2. By deepening the number of convolutional layers, the CNN can gradually extract higher-level features which are robust and discriminative for the epileptic seizure recognition tasks.

### LSTM Structure

**FIGURE 3:**
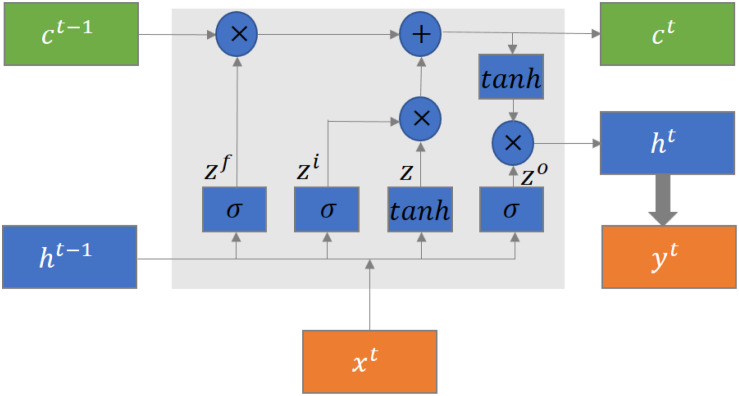
The typical structure of an LSTM block.

The typical structure of an LSTM block is shown in Figure 3 (Yuan et al., 2020). There are four gates in the LSTM block, which are cell state gate z which remembers the information over time, forget gate z^*f*^ which controls the extent of the value kept in the cell, input gate z^*i*^ which controls the extent of the value flow in the cell, and output gate z^*o*^ which controls the extent of the value in the cell to be used for computing the output. Each gate contains a fully connected layer and an activation function. In addition, there are three inputs, which are cell state c^*t*−1^, previously hidden state h^*t*−1^, and current input x^*t*^, and three outputs, which are cell state c^*t*^, hidden state h^*t*−1^, and current output y^*t*^, in the LSTM block. The current output is generated based on the hidden state. The mathematical formulation of the LSTM units is defined as follows:

$$z^{f}=\sigma \,\left(W^{f}\,\left[x_{t},h_{t-1}\right]\right)$$

$$z^{i}=\sigma \,\left(W^{i}\,\left[x_{t},h_{t-1}\right]\right)$$

$$z=t\,a\,n\,h\,\left(W\,\left[x_{t},h_{t-1}\right]\right)$$

$$z^{o}=\sigma \,\left(W^{o}\,\left[x_{t},h_{t-1}\right]\right)$$

$$c^{t}=z^{f}\times c^{t-1}+z^{i}\times z$$

$$h^{t}=z^{o}\times \tanh\left(c^{t}\right)$$

$$y^{t}=\sigma \,\left(W^{\prime }\,h_{t}\right)$$

### 1D CNN-LSTM Model

**FIGURE 4:**
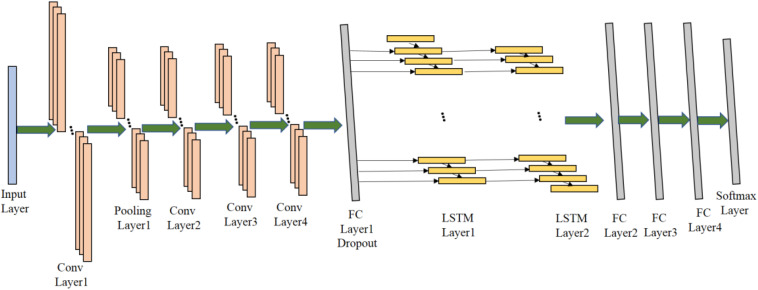
The structure of the 1D CNN-LSTM model.

The proposed 1D CNN-LSTM model is composed of an input layer, four convolutional layers, one pooling layer, two LSTM layers, four fully connected (FC) layers, and a soft-max output layer. The detailed network structure is shown in Figure 4 (Jiang et al., 2015; Zhao et al., 2019; Cura et al., 2020).

Firstly, the 1D EEG signal data is directly used as the input of the proposed model, and the shape of the input data is 178 × 1. Then, the input data is passed through the first convolutional layer in order to extract abstract features of raw signal data, where the number of 1D convolutional kernels in the Conv Layer1 is 64, the shape of each convolutional kernel is 3 × 1 and the stride of convolutional kernels is 1. This convolutional layer is followed by a Rectified Linear Unit (ReLU) activation layer which can introduce non-linearity to the proposed model. Here, the mathematical definition of the 1D convolutional operation and the ReLU activation is described as follows:

$$y_{j}^{l}=\sigma \,\left(\sum_{i=1}^{N_{l-1}}c\,o\,n\,v\,1\,D\,\left(w_{i,j}^{l},x_{i}^{l-1}\right)+b_{j}^{l}\right)$$

where $x_{i}^{l-1}$ represents the *i*th feature map in the (*l-1*)th layer; $y_{j}^{l}$ represents the *j*th feature map in the *l*th layer; $w_{i,j}^{l}$ represents the trainable convolutional kernel; *N*_*l–1*_ represents the number of feature maps in the (l-1)th layer; *conv1D* represents the 1D convolution operation without zero-padding, therefore, the dimension of the feature map in the *l*th layer is less than that in the *l-1*th layer; $b_{j}^{l}$ represents the bias of the *j*th feature map in the *l*th layer; σ() represents the ReLU activation function, which can help avoid the overfitting problem. It is defined as follows:

$$\sigma \,\left(x\right)=\left\{ \begin{array}{cc} 0, & x\leq 0\\ x, & x>0 \end{array}\right.$$

After the convolution and activation, 64 feature maps with the size of 176 × 1 are outputted. After that, the output of the Conv Layer1 is then passed through a max-pooling layer. Here, the mathematical definition of the 1D max-pooling operation is described as follows:

$$p_{i}^{a}=\text{max}\left(p_{i}^{a^{\prime }}:a\leq a^{\prime }<a+s\right)$$

where $p_{i}^{a^{\prime }}$ is the *a*′th neuron in the *i*th feature map before max-pooling operation and $p_{i}^{a}$ is the *a*th neuron in the *i*th feature map after max-pooling operation, and *s* is the size of pooling window. In the Pooling Layer1, the size of pooling window is 2 and the stride of windows is also 2. It can significantly reduce the number of training parameters in the proposed model and accelerate the training process. After the pooling operation, 64 feature maps with the size of 88 × 1 are outputted. Then, three convolutional layers are followed to further extract higher-level features which can facilitate the classification. They are Conv Layer2, Conv Layer3, Conv Layer4, there are 128 kernels in the shape of 3 × 1 in the Conv Layer 2, 512 kernels in the same shape in the Conv Layer3, and 1024 kernels in the same shape in the Conv Layer4. Similarly, the convolution operation is the same as that in the Conv Layer1, and ReLU is also applied for non-linear activation.

After the feature maps passing through all the 1D convolutional layers, the obtained 1024 feature maps with the size of 82 × 1 will be fed into one FC layer with 256 neurons and dropout is then applied to the output of the FC layer. FC Layer1 can concatenate the output from the convolution layers and reduce the dimension of feature maps in order to fit the input of LSTM layers, and dropout can alleviate the overfitting concerns to some extent.

After passing through the FC Layer1, the output features are fed into the LSTM layers which is capable of avoiding the long-term dependency problem in the standard RNN. There are four gates including the cell state gate, forget gate, input data, output gate, in the LSTM cell. They can collaborate with each other to preserve the previous information and further improve the ability of learning useful information from EEG time-series data. There are 64 neurons in both the LSTM Layer1 and the LSTM Layer2.

After the features passing through the LSTM layers, the output features will then be fed into three FC layers. FC Layer2, FC Layer3 and FC Layer4 are fully connected layers with 256, 128, and 64 neurons, respectively. Finally, a softmax output layer is added to the proposed model for final recognition. The detailed configuration of the proposed model can be adjusted according to the specific epileptic seizure recognition task.

### Model Application

After the proposed model is successfully built and trained, the 1D CNN-LSTM model is applied to recognize epileptic seizure activity.

## Method Evaluation

In this section, the performance of the proposed method is evaluated by the experiments conducted on the public UCI epileptic seizure recognition data set and the training and testing results of the proposed method are given. Additionally, comparative experimental results with traditional machine learning methods and other deep learning methods are also given in order to show its superiority. All the experiments are conducted on a deep learning workstation equipped with an Intel 12-core 3.5-GHz CPU, a GTX1080TI GPU, 256 GB SSD, and 96 GB Memory.

### Experimental Setup

During the whole process of the experiment, we selected 90% of the data as the training set and 10% of the data as the test set. As for the deep neural network (DNN), CNN and, 1D convolutional LSTM models, the number of training epochs is set as 100. In order to improve the generalization performance and avoid the overfitting problem, the dropout technique is used in the proposed method.

It should be pointed out that the data are randomly shuffled before training and then fed into the network. During training, at the end of each epoch, the accuracy of the proposed 1D CNN-LSTM model on the training data set and test data set are both calculated, which can help us to judge whether the model is overfitting and thereby verify the generalization ability of the current model. In addition, we add checkpoints during the training process, if the generalization ability of the model has not improved within the 10 training processes, the learning rate will be changed.

Two types of epileptic seizure recognition tasks are considered in this paper, namely binary and five-class recognition tasks. To be specific, the epileptic seizure condition and the normal condition are included in the binary task, and the epileptic seizure condition and the four normal conditions, including eyes open, eyes closed, EEG activity from the healthy brain area, and EEG activity from the tumor area, are included in the five-class task.

### Experimental Results and Analysis

#### Binary Recognition Task

**FIGURE 5:**
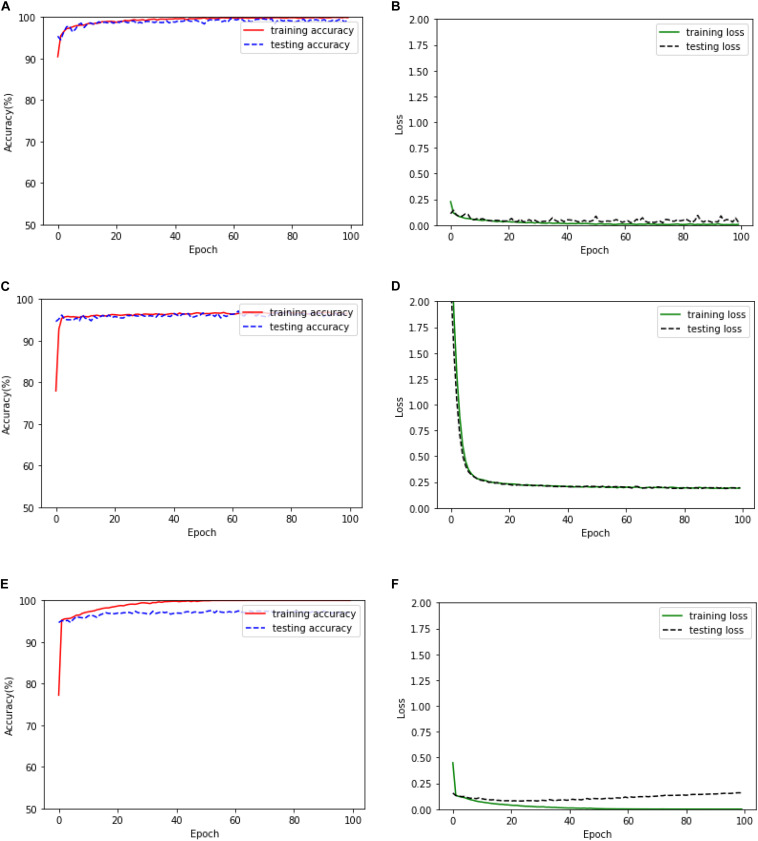
Training and testing results of the binary recognition task. **(A)** The training and test accuracy of the proposed model. **(B)** The training and test loss of the proposed model. **(C)** The training and test accuracy of the DNN. **(D)** The training and test loss of the DNN. **(E)** The training and test accuracy of the standard CNN. **(F)** The training and test loss of the standard CNN.

In this subsection, firstly, the training accuracy, testing accuracy, training loss, and testing loss of the proposed method based on the 1D CNN-LSTM model when it is applied to the binary recognition task is given in Figure 5. In addition, two deep learning models are also realized for epileptic seizure recognition in order to compare them with the proposed model, which are the DNN and standard CNN. The training and testing results of the DNN and the standard CNN are also given in Figure 5.

It can be seen from Figure 5 that the DNN has the fastest convergence speed, while the training loss and testing loss of the proposed model decrease at the lowest speed. Therefore, the proposed model needs more training time. On the other hand, the training and testing loss values of the proposed model are obviously smaller than the values of the DNN model, thus obtaining better training and testing accuracies. However, although the training performance of the standard CNN is similar to that of the proposed model, the testing performance of the standard CNN degrades seriously after the early stage of the training process, which is significantly inferior to the proposed model.

**FIGURE 6:**
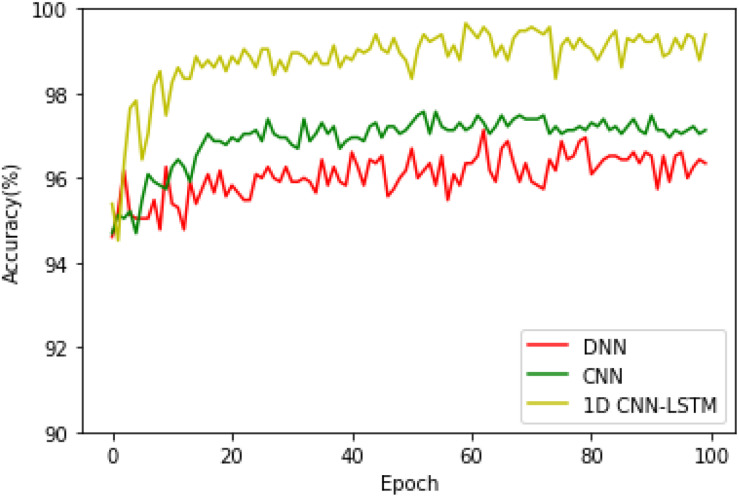
Testing accuracies of these three models on the binary recognition task.

After that, in order to see the accuracy superiority of the proposed 1D CNN-LSTM model over the DNN and CNN models in more detail, the testing accuracies of these three models on the binary recognition tasks are shown in Figure 6. It can be seen from Figure 6 that the proposed model achieves the highest testing accuracy throughout most of the training process. Therefore, it can be concluded that the proposed model is superior to the DNN model and standard CNN model in both the training and testing processes.

Finally, in order to further evaluate the seizure classification performance of these three models, the accuracy, precision, recall, and F1-score metrics are calculated and compared in Table 1. These performance metrics are briefly introduced as follows:

$$\text{Accuracy}=\frac{T\,P+T\,N}{T\,P+T\,N+F\,P+F\,N}$$

$$\text{Precision}=\frac{T\,P}{T\,P+F\,P}$$

$$\text{Recall}=\frac{T\,P}{T\,P+F\,N}$$

$$\text{F}\,1-\text{Score}=2\times \frac{\text{Precision}\times \text{Recall}}{\text{Precision}+\text{Recall}}$$

**TABLE 1:** The performance of DNN, CNN, and the proposed 1D CNN-LSTM model on the binary classification task.

Methods	Accuracy	Precision	Recall	F1-score
DNN	96.35%	95.18%	87.50%	0.9118
CNN	97.13%	94.24%	92.34%	0.9328
Proposed model	99.39%	98.39%	98.79%	0.9859

where the values of *TP* and *FN* represent the number of a given seizure recognition task being correctly classified and incorrectly classified, respectively; *TN* represents the number of seizure recognition tasks not belonging to a given class not being classified as this class; *FP* represents the number of a given seizure recognition task being incorrectly classified as this type.

It can be seen from Table 1 that the accuracy, precision, recall, and the F1-score of the proposed model are 99.39%, 98.39%, 98.79%, and 0.9859%, respectively, which is significantly better than the DNN and standard CNN models. To be specific, compared with the DNN model and the standard CNN model, the proposed model obtains accuracy improvements of 3.04% and 2.26%, precision improvements of 3.21% and 4.15%, recall improvements of 11.29% and 6.45%, and F1-score improvements of 0.074 and 0.053.

#### Five-Class Recognition Task

**FIGURE 7:**
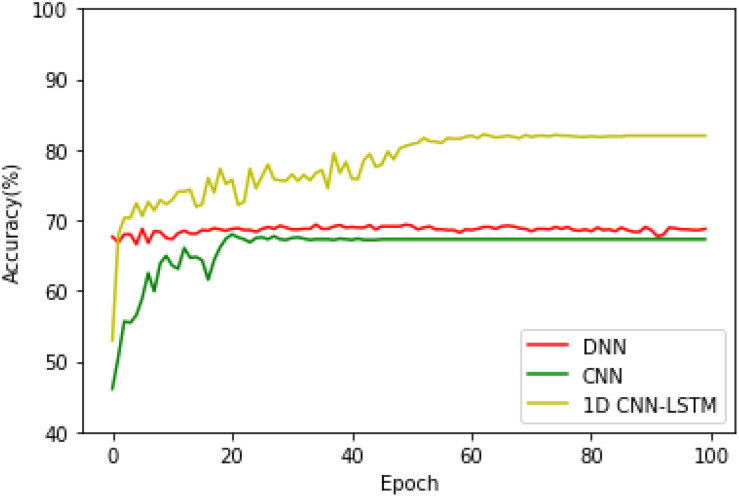
Testing accuracies of the three models on the five-class recognition task.

Then, the training and testing processes of the above-mentioned three models when they are applied to the five-class recognition task are conducted, and the testing accuracies of these three models are given in Figure 7. It can be found that the DNN and CNN models exhibit a similar accuracy performance, while the proposed 1D CNN-LSTM model obtains the best recognition performance regardless of the different recognition tasks.

Table 2 provides the accuracy, precision, recall, and F1-score of the DNN, CNN, and the proposed model. From Table 2, it can be seen that the proposed model significantly outperforms the DNN and CNN models in terms of all the metrics.

**TABLE 2:** The performance of DNN, CNN, and the proposed 1D CNN-LSTM model on the five-class classification task.

Methods	Accuracy	Precision	Recall	F1-score
DNN	68.78%	67.63%	67.67%	0.6691
CNN	67.30%	67.73%	66.76%	0.6705
Proposed model	82.00%	81.78%	81.70%	0.8156

#### Additional Analysis

In this subsection, two additional analysis experiments are conducted. The first one is to determine the most suitable number of neurons in the hidden LSTM layer. Therefore, the number of neurons in the hidden LSTM layer is changed. The accuracy comparison results of the proposed 1D CNN-LSTM model with 32, 64, 128, 192, and 256 neurons in the hidden LSTM layer are given in Table 3.

**TABLE 3:** Test accuracies of the proposed 1D CNN-LSTM model with different numbers of neurons in the hidden LSTM layer.

The number of neurons in the hidden LSTM layer	32	64	128	192	256
Accuracy on binary recognition task	99.22%	99.39%	99.04%	99.39%	99.39%
Accuracy on five-class recognition task	80.78%	82.00%	79.48%	80.70%	78.61%

It can be found that the when the number of neurons in the LSTM layer equals 64, the test accuracy of the proposed model reaches 99.39% on the binary recognition task, which is higher than others. On the other hand, when the number of neurons in the LSTM layer equals 64, the best accuracy of 82.00% is achieved on the five-class recognition task.

**FIGURE 8:**
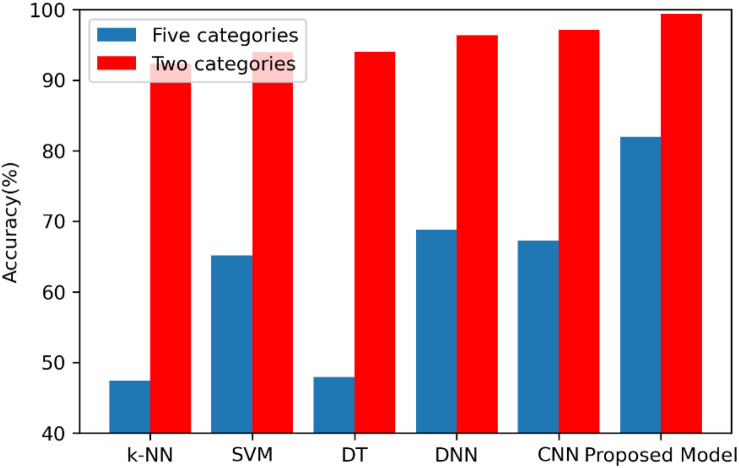
Average accuracies of the k-NN, SVM, DT, DNN, CNN, and 1D CNN-LSTM models.

The second analysis experiment is to further verify the accuracy superiority of the proposed model. In this paper, apart from the deep learning model, the following three machine learning-based approaches are also realized to compare them with the proposed method in order to further test the superiority of the proposed model, which are k-nearest neighbor (k-NN), decision tree (DT), and support vector machine (SVM) models. All the experiments of these models are carried out 10 times, and the average accuracies of the k-NN, SVM, DT, DNN, CNN, and 1D CNN-LSTM models are shown in Figure 8.

On the binary recognition task, the accuracy of the proposed model is 99.39%, which is 7.09% higher than k-NN, 5.43% higher than SVM, and 5.35% higher than DT. On the five-class recognition task, the accuracy of the proposed model is 82.00%, which is 34.57% higher than k-NN, 16.87% higher than SVM, and 34.05% higher than DT. All these results prove the strong potential of the proposed 1D CNN-LSTM model in the research area of EEG-based epileptic seizure recognition.

## Conclusion and Future Work

In this paper, a 1D CNN-LSTM model is proposed for epileptic seizure recognition through EEG signal analysis. The proposed model combines a 1D CNN and an LSTM to construct an end-to-end network that can accurately classify normal and epileptic seizure EEG signals. The 1D CNN has the strong ability of EEG signal feature extraction and the LSTM network is able to memorize and recognize the sequential EEG signals. The performance of the proposed model is verified by the experiments conducted on the well-known UCI epileptic seizure recognition data set. Two epileptic seizure recognition tasks, including binary and five-class recognition tasks, are carried out. The proposed model achieves high accuracies of 99.39% and 82.00% on the two tasks, respectively. In addition, taking the binary recognition task for example, the proposed model achieves accuracy improvements of 3.04%, 2.26%, 7.09%, 5.43%, and 5.35% compared with the other methods including DNN, CNN, k-NN, SVM, and DT, respectively.

Although the proposed model has achieved considerable progress in the area of epileptic seizure recognition, there are still two limitations that need to be further addressed in the future. First, the recognition performance of the proposed model on the multi-class tasks is not very satisfying. Second, the supervised training of the proposed model needs a large amount of labeled EEG signal data. However, collecting sufficient labeled data is time-consuming and laborious. Based on these limitations, the future work will focus on two aspects: first, the proposed model can be further modified and optimized in order to improve its performance on the more complex epileptic seizure recognition tasks, which will improve its classification ability on different data sets; second, the transfer learning technique can be introduced to the proposed model in order to alleviate the dependence on the labeled signal data.

## Data Availability Statement

Publicly available datasets were analyzed in this study. This data can be found here: https://archive.ics.uci.edu/ml/datasets/Epileptic+Seizure+Recognition.

## Author Contributions

GX supervised this research and revised the manuscript. TR conducted the experiments and drafted the manuscript writing. YC and WC checked for grammatical errors of the manuscript and discussed difficult problems in the manuscript with GX. All authors contributed to the article and approved the submitted version.

## Conflict of Interest

The authors declare that the research was conducted in the absence of any commercial or financial relationships that could be construed as a potential conflict of interest.
